# Association of white matter hyperintensities with long‐term EGFR‐TKI treatment and prediction of progression risk

**DOI:** 10.1002/brb3.3326

**Published:** 2023-12-06

**Authors:** Hang Yang, Rui Meng, Junjie Jiang, Yan Luo, Xiaolin Deng, Sibo Yang, Shengcai Chen, Jiehong Wu, Yan Wan, Yanan Li, Huijuan Jin, Quanwei He, David Wang, Jiang Chang, Kunyu Yang, Yifan Zhou, Bo Hu

**Affiliations:** ^1^ Department of Neurology Union Hospital, Tongji Medical College Huazhong University of Science and Technology Wuhan China; ^2^ Cancer Center, Union Hospital Tongji Medical College Huazhong University of Science and Technology Wuhan China; ^3^ Neurovascular Division Department of Neurology Barrow Neurological Institute St. Joseph's Hospital and Medical Center Phoenix Arizona USA; ^4^ Department of Epidemiology and Biostatistics, Key Laboratory for Environment and Health, School of Public Health, Tongji Medical College Huazhong University of Science and Technology Wuhan China

**Keywords:** epidermal growth receptor‐tyrosine kinase inhibitor, non‐small cell lung cancer, white matter hyperintensities

## Abstract

**Purpose:**

The purpose of this study was to test the hypothesis that brain white matter hyperintensities (WMH) are more common in patients receiving epidermal growth factor receptor tyrosine kinase inhibitor (EGFR‐TKI) and identify clinical risk factors associated with WMH.

**Experimental design:**

This multiple‐center, prospective cohort study was conducted from March 2017 to July 2020. Two groups of patients with non‐small cell lung cancer (NSCLC) who received or did not receive EGFR‐TKI were included and followed up for more than 24 months. The progression of WMH was defined as an increase of ≥1 point on the Fazekas visual rating scale between the baseline and at the 2‐year follow‐up. A modified Poisson regression model was performed to evaluate risk factors on increased WMH load.

**Results:**

Among 286 patients with NSCLC, 194 (68%) patients with NSCLC who received EGFR‐TKI and 92 (32%) patients with NSCLC without EGFR‐TKI treatment were analyzed. Modified Poisson regression analysis showed that EGFR‐TKI treatment was independently associated with the WMH progression (EGFR‐TKI: aRR 2.72, 95% confidence interval [CI] 1.46–5.06, *p* = .002). Interleukin (IL)‐2, IL‐4, and IL‐10 were associated with increased WMH in the adjusted model (IL‐2: aRR 1.55 [95% CI 1.06–2.25], *p* = .023; IL‐4: aRR 1.66 [95% CI 1.13–2.43], *p* = .010; IL‐10: aRR 1.48 [95% CI 1.06–2.06], *p* = .020).

**Conclusion:**

Patients with NSCLC who received EGFR‐TKI may be at higher risk of developing WMH or worsening of WMH burden. The impact of increased WMH lesions in these patients is to be further assessed. IL‐2, IL‐4, and IL‐10 may be used as potential biomarkers to monitor the risk of increased WMH burden

## Graphical abstract

This study suggested an association of treated with EGFR‐TKI, IL‐2, IL‐4 and IL‐10 with the progression of white matter hyperintensities (WMH). By analyzing transcriptomic data, genes enriched in on signaling pathways such as MAPK, suggesting a possible mechanism of WMH progression.

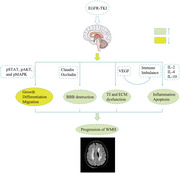

## 1 INTRODUCTION

The treatment of non‐small cell lung cancer (NSCLC) is “individualized” with targeted drugs. This approach is more effective, has fewer side effects, and is gradually replacing traditional chemotherapy (Ettinger et al., 2016; Wu et al., 2019). Epidermal growth factor receptor (EGFR) mutation is the most common agent used to target the mutation in NSCLC (Holleman et al., 2019; Loong et al., 2018). Therefore, the use of epidermal growth factor receptor tyrosine kinase inhibitor (EGFR‐TKI) is one of the research hotspots in lung cancer treatment.

Many studies have found that epidermal growth factor (EGF) promotes cell proliferation, regeneration, neuronal development, and injury repair (Furnari et al., 2015; Goldshmit et al., 2004; I.‐S. Kim et al., 2017). Therefore, the inhibition of the EGF pathway or anti‐EGFR therapy by EGFR‐TKI may have a negative impact on neuronal differentiation, maturation, and recovery. The common adverse effect of anti‐EGFR therapy includes skin toxicities (31.4%), diarrhea (14.2%), pruritus (6.7%), and hepatic toxicity (3.8%) (Shah & Shah, 2019). However, studies have also reported that patients with NSCLC treated with long‐term EGFR‐TKI have developed mental sluggishness, memory deterioration, and cognitive disorder. (Kang et al., 2019; Zhu et al., 2020). These symptoms are related to the development of brain white matter lesions that are insidious. White matter lesions appear as white matter hyperintensities (WMH) on magnetic resonance imaging (MRI) T2 images (Martorell et al., 2012). They are often associated with older age, cerebrovascular diseases, and dementia (Erten‐Lyons et al., 2013; Young et al., 2008). Some studies have reported that targeted drugs might induce cognitive and psychiatric problems in patients with NSCLC. However, increased WMH was found in patients treated with EGFR‐TKI during our routine follow‐ups. In this study, we investigated EGFR‐TKI treatment with WMH load and explored the possible mechanisms of increased WMH load in patients with NSCLC receiving EGFR‐TKI.

## 2 METHODS

### 2.1 Participants

This study was approved by the Medical Ethics Committee of Tongji Medical College, Huazhong University of Science and Technology, Wuhan, China. Consecutive patients with NSCLC were screened for eligibility for the ongoing prospective cohort study from March 2017 to July 2020. Three clinical centers (Main District of Wuhan Union Hospital, Cancer Center of Wuhan Union Hospital, and Wuhan Union Hospital West Branch) are responsible for recruitment, enrolment, and protocol implementation. Patients with EGFR mutation were required to be on EGFR‐TKI therapy for at least 1 year. All patients were required to return to the outpatient clinic at least once every 3 months. An annual brain MRI is performed for patients who do not have neurological symptoms. All patients were followed up for at least 2 years. Exclusion criteria were (1) the presence of comorbidities affecting the white matter structure of the brain and (2) contraindication to MRI scanning. The following data were obtained at interviews on‐site and through a review of medical records: gender, age, weight, height, past medical history, medication use, history of brain metastasis, cardiovascular risk factors, clinical examination, laboratory examination, and imaging examination. Patients who used immune checkpoint inhibitors were not included in the analysis.

### 2.2 Image acquisition and processing

All participants underwent baseline and follow‐up MRI scans. MRI images from the baseline and second years were analyzed. We collected the following brain MRI sequences: T1‐weighted (TR/TE = 2500/24 ms), T2‐weighted (TR/TE = 5100/130 ms), and T2‐FLAIR (fluid‐attenuated inversion recovery, TR/TE = 9000/120 ms). WMH were evaluated on T2‐FLAIR images and manually rated using the Fazekas visual rating scale, with a score of 0−3 for periventricular hyperintensities (0 = no lesions, 1 = pencil‐thin lining, 2 = smooth halo, and 3 = irregular with extension into deep white matter) and 0–3 for deep WMH (0 = no lesions, 1 = punctate foci, 2 = beginning confluence of foci, and 3 = large confluent areas) (Fazekas et al., 1987). Two neurologists manually outlined the region of WMH by the 3D Slicer software (4.11 version; http://www. slicer.org) and rated independently, blinded for clinical data. We use the painting in the software to manually draw the WMH area to obtain the volume. Since the baseline WMH volume is related to intracranial volume (ICV), we calculated the percentage of WMH volume by dividing the ICV. Slicer segmentations of ICV were created using a thresholding tool and specialized segmentation extension SwissSkullStripper with minimal use of manual tools such as painting and erasing. It is important to normalize the head MRI scans of various persons in order to create the WMH distribution map. WMH maps were nonlinearly aligned to the T2 template provided by Advanced Normalization Tools (https://github.com/ANTsX/ANTs) (Tustison et al., 2014). Two neurologists (Yifan Zhou and Hang Yang) assessed a randomly selected sample of MRIs to determine intra‐ and interrater reliability.

### 2.3 Outcome measures

WMH progression was defined as a one‐point or greater increase on the Fazekas visual rating scale between baseline and follow‐up.

### 2.4 Statistical analysis

We first compared the data distribution of each covariate among the groups, using the *t*‐test (normal distribution) or Kruskal–Wallis rank sum test (non‐normal distribution) for continuous variables and chi‐square tests for categorical data. The effect values and statistical significance were then assessed using modified Poisson regression without and with adjustments for sex, age, and body mass index (BMI). Inter‐ and intrarater reliability for the Fazekas scale was assessed using Dice kappa. All analyses were performed with R (http://www.R‐project.org) and the EmpowerStats software (X&Y Solutions, Inc.; www.empowerstats.com). A significance level of 0.05 and a power of 0.9 were used for all statistical tests.

### 2.5 Identification of common pathways and biological processes

The following transcriptome data from the GEO database were used to study (https://www.ncbi.nlm.nih.gov/geo/): PC9 cells were treated for 2 weeks with 300 nM Gefitinib (GSE114647), and periventricular white matter lesions were extracted from frozen human post‐mortem CNS tissue (GSE157363) (Fadul et al., 2020; Raoof et al., 2019). Quantile normalization and log2 transformation were employed for data pre‐processing with R software. Differentially expressed genes (DEGs) were performed using functions in the limma package (version 3.20.1). Genes with an adjusted *p*‐value <.05 and |log fold change| (|log FC|) > 1 were assigned as DEGs in both datasets. Gene ontology (GO) biological process and Kyoto encyclopedia of genes and genomes (KEGG) pathway enrichment analysis of these significant DEGs were performed by Metascape (https://metascape.org) with a *p*‐value cutoff of .01, a minimum enrichment of 1.5 and a minimum overlap of 3 (Zhou et al., 2019). The heatmap of overlapping DEGs at the common KEGG pathway was generated using the TBtools software (https://github.com/CJ‐Chen/TBtools) (Chen et al., 2020).

## 3 RESULTS

### 3.1 Patient characteristics

Between March 1, 2017, and July 30, 2020, we screened a total of 537 patients with NSCLC. Among them, 322 were treated with EGFR‐TKI, and 215 received no EGFR‐TKI. A total of 199 patients had missing follow‐up images or inadequate/missing MRI sequences; 39 had missing baseline imaging and 13 had incomplete clinical information. Hence, statistical analyses were conducted on 286 patients (194 in the EGFR‐TKI group and 92 in the non‐EGFR‐TKI group). Among the 286 patients with NSCLC, the mean age was 59.1 (SD, 9.5) years, and 62% were male. Clinical and demographic comparisons between the groups are presented in Table 1. The inter‐ and interrater reliability was high (intrarater reliability: 0.92 and 0.87; interrater reliability: 0.83).

**TABLE 1:** Baseline clinical and laboratory characteristics of the patients.

Variable	Total (N = 286)	EGFR‐TKI (N = 194)	Non‐EGFR‐TKI (N = 92)	*p*‐value
Age, years	59.1 ± 9.5	58.0 ± 9.7	61.4 ± 8.6	.005
Sex, male	149 (62)	78 (40)	70 (76)	<.001
BMI, kg/m^2^	23.0 ± 3.0	23.1 ± 3.1	22.9 ± 2.8	.637
Hyperlipidemia	48 (17)	39 (20)	8 (9)	.014
Hypertension	49 (17)	35 (18)	14 (15)	.542
Diabetes mellitus	30 (10)	20 (10)	10 (11)	.896
History of smoke	78 (27)	36 (19)	41 (45)	<.001
Heavy drinking	23 (8)	8 (4)	15 (16)	<.001
Brain metastases	90 (31)	71 (37)	17 (18)	.002
Brain radiotherapy	69 (24)	54 (28)	15 (16)	.033
Systolic blood pressure, mmHg	125 ± 14	125 ± 14	123 ± 13	.214
Diastolic blood pressure, mmHg	78 ± 9	78 ± 10	78 ± 9	.565
IL‐2, pg/mL	2.16 (1.12–3.51)	2.48 (1.23–3.54)	1.64 (1.10–3.17)	.211
IL‐4, pg/mL	2.00 (1.08–2.94)	2.42 (1.02–3.12)	1.73 (1.29–2.60)	.501
IL‐6, pg/mL	7.59 (4.73–19.88)	7.00 (4.55–21.66)	8.46 (4.78–18.52)	.469
IL‐10, pg/mL	3.21 (1.27–4.30)	3.31 (1.11–4.43)	3.12 (1.85–3.91)	.963
CEA, ng/mL	8.7 (3.16–28.90)	12.33 (4.35–41.33)	3.74 (2.56–15.50)	.103
CA19‐9, ng/mL	9.60 (3.50–32.6)	9.80 (4.20–37.70)	6.80 (3.20–25.20)	.378
EFGR‐TKI				
First generation	158 (55)	158 (81)	–	–
Second and third generation	11 (4)	11 (6)	–	–
Third generation	25 (9)	25 (13)	–	–
WMH progression	60 (21)	49 (25)	11 (12)	0.006
WMH volume of baseline, mL	0.74 (0.21–2.64)	0.63 (0.14–2.36)	0.97 (0.29–3.25)	0.315
WMH annual growth volume, mL/year	2.53 ± 9.14	2.70 ± 9.81	2.27 ± 8.18	0.048

*Note*: Data presented as mean ± SD, *n* (%) or median (25th−75th percentiles). *p*‐value indicates the comparison between the group taking EGFR‐TKI and the non‐EGFR‐TKI group.

Abbreviations: BMI, body mass index; CA19‐9, carbohydrate antigen 19‐9; CEA, carcinoembryonic antigen; EGFR, epidermal growth factor receptor; IL, interleukin; TKI, tyrosine kinase inhibitor; WMH, white matter hyperintensities.

### 3.2 WMH differences between groups

**FIGURE 1:**
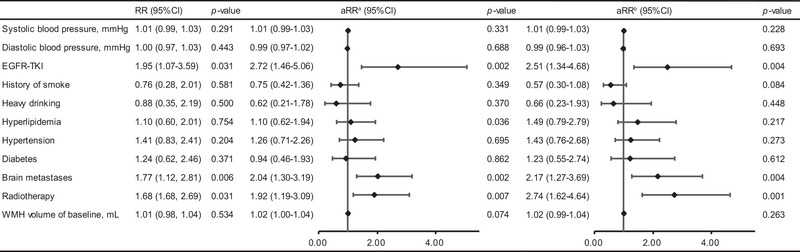
Associations between clinical risk factor variables and increased white matter hyperintensities (WMH) Load. ^a^Adjust for sex, age, and BMI. ^b^Adjust for sex, age, and BMI, baseline Fazeka score. BMI, body mass index; CI, confidence interval; EGFR, epidermal growth factor receptor; TKI, tyrosine kinase inhibitor.

**FIGURE 2:**
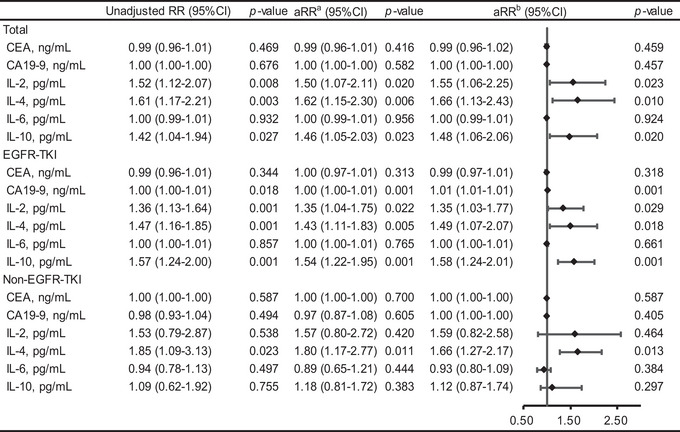
Associations between laboratory biomarker following epidermal growth factor receptor tyrosine kinase inhibitor (EGFR‐TKI) treatment and white matter hyperintensities (WMH). ^a^Adjust for sex, age, and BMI. ^b^Adjust for sex, age, BMI, hyperlipidemia, hypertension, and diabetes. BMI, body mass index; CEA, carcinoembryonic antigen; CA19‐9, carbohydrate antigen 19‐9; IL, interleukin.

Clinical variables were obtained for the risk factor of WMH progression. The modified Poisson regression analysis with the adjustment of sex, age, and BMI showed that patients who received EFGR‐TKI treatment had a 2.72‐fold higher relative risk (aRR 2.72 [95% confidence interval [CI] 1.46−5.06], *p* = .002; aRR^b^ 2.51 [95% CI 1.34−4.68], *p* = .004) of increased WMH load (Figure 1). Patients with brain metastases were significantly more likely to develop white matter progression (aRR^a^ 2.04 [95% CI 1.30−3.19], *p* = .002; aRR^b^ 2.17 [95% CI 1.27−3.69], *p* = .004). Receiving brain radiation therapy was also a risk factor for white matter progression (aRR^a^ 1.92 [95% CI 1.19−3.09], *p* = .007; aRR^b^ 2.74 [95% CI 1.62−4.64], *p* = .001). Estimation after controlling the history of smoking and heavy drinking generated similar results (Table S1). Laboratory indexes interleukin [IL]‐2, IL‐4, and IL‐10 were positively correlated with WMH load (Figure 2). IL‐2, IL‐4, and IL‐10 levels remained to be an independent predictor of WMH progression in the adjusted model (IL‐2: aRR 1.55 [95% CI 1.06−2.25], *p* = .023; IL‐4: aRR 1.66 [95% CI 1.13−2.43], *p* = .010; IL‐10: aRR 1.48 [95% CI 1.06−2.06], *p* = .020). Analysis of those receiving EGFR‐TKI therapy showed similar results (Figure 2). We reported effect size between laboratory biomarkers and WMH in Table S2).

### 3.3 WMH differences between the EGFR‐TKI group and non‐EGFR‐TKI group

**FIGURE 3:**
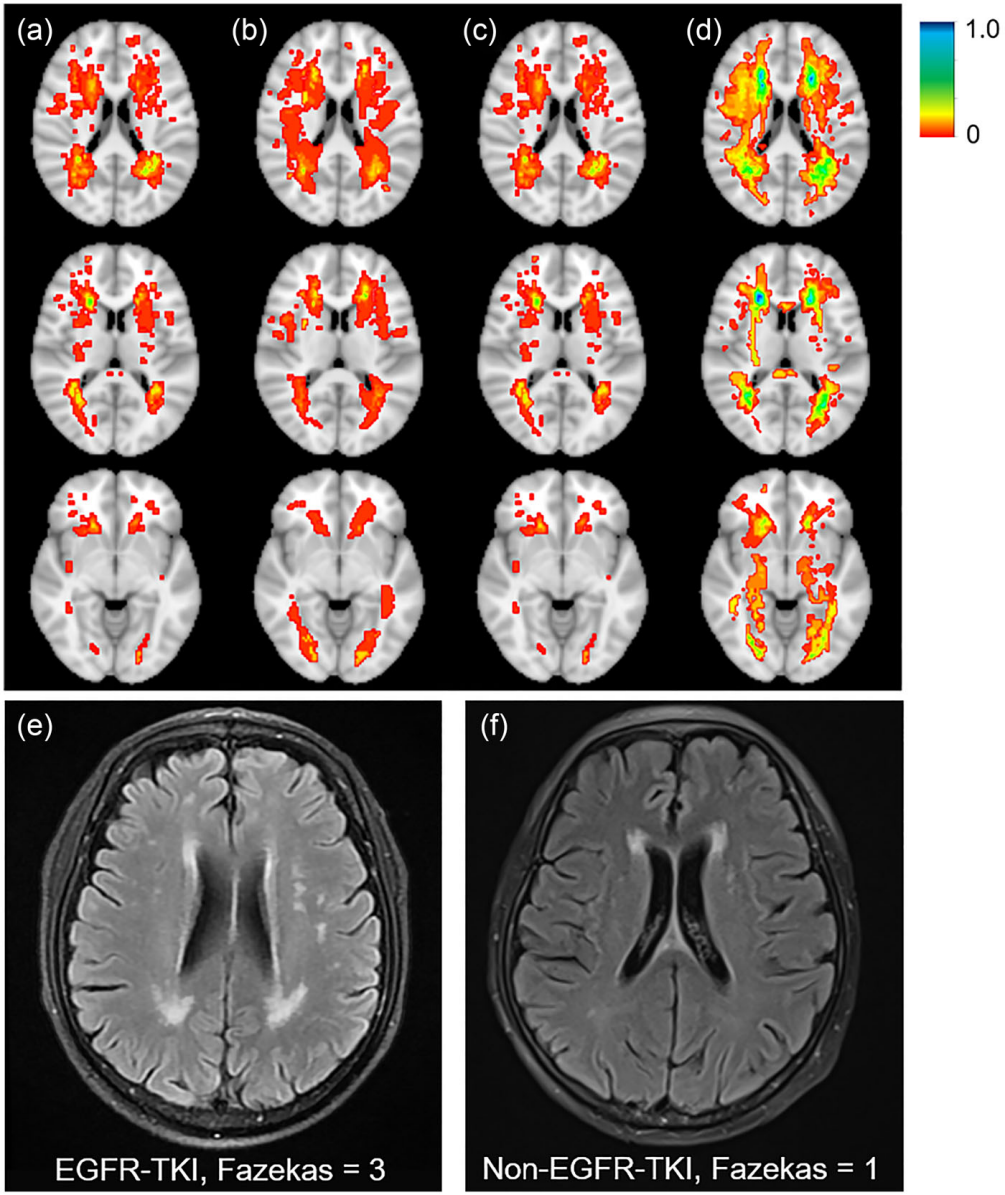
The dynamic changes of white matter hyperintensities white matter hyperintensities (WMH). (a) Spatial distribution of baseline WMH in patients who had not received epidermal growth factor receptor tyrosine kinase inhibitor (EGFR‐TKI) treatment. (b) WMH in the patient had not received EGFR‐TKI at the 2‐year follow‐up. (c) Baseline WMH in patients had received EGFR‐TKI. (d) WMH in the patient had received EGFR‐TKI at the 2‐year follow‐up. T2 Flair images from magnetic resonance imaging (MRI) scans of (e) a 64‐year‐old man with non‐small cell lung cancer (NSCLC) taking EGFR‐TKI 1 year before the scan, a total Fazekas score of 3 (2 for periventricular and 1 for deep) and (f) a 61‐year‐old man has not received EGFR‐TKI with NSCLC, no known vascular risk factors, with a total Fazekas score of 1 (1 for periventricular and 0 for deep).

The proportions of raw scores (total Fazekas score, periventricular WMH score, and deep WMH score) for each group are presented in Figure S1, and it was evident that the EGFR‐TKI group had higher scores in the total and periventricular than the non‐EGFR‐TKI group. Data on the distribution of Fazekas scores change revealed that the EGFR‐TKI group had more participants with a progression of two points or more (Table 2). Figure 3 illustrates the spatial distribution of WMH in the non‐EGFR‐TKI and received EGFR‐TKI treatment groups. WMH in the patient who had received EGFR‐TKI involved more of the periventricular and deep white matter.

**TABLE 2:** Distribution of Fazekas scores change in white matter hyperintensities progressed patient.

Fazekas score change	EGFR‐TKI (*n*)	Non‐EGFR‐TKI (*n*)	*p*‐value
0–1	11	3	.004
0–2	1	1	
0–3	2	0	
1–2	22	5	
1–3	2	1	
2–3	7	1	

Abbreviations: EGFR, epidermal growth factor receptor; TKI, tyrosine kinase inhibitor.

### 3.4 Subgroup analysis

Among patients with EGFR‐TKI treatment, hyperlipidemia and radiation therapy were associated with increased WMH (hyperlipidemia: aRR 1.65 [95% CI 1.01−2.70], *p* = .047; radiation therapy: aRR 2.37 [95% CI 1.51−3.71], *p* = .010). IL‐2, IL‐4, and IL‐10 levels independently predicted WMH progression in the adjusted model for the EGFR‐TKI group (IL‐2: aRR 1.30 [95% CI 1.10−1.53], *p* = .002; IL‐4: aRR 1.43 [95% CI 1.14−1.81], *p* = .003; IL‐10: aRR 1.31 [95% CI 1.10−1.60], *p* = .003) (Table 3). Further analysis of non‐metastases and metastases subgroups showed that SBP was associated with the WHM progression for those in the non‐metastases group (aRR 1.02 [95% CI 1.00−1.04], *p* = .020).

**TABLE 3:** Risk factor and laboratory biomarker analysis for increased white matter hyperintensities load in subgroup of epidermal growth factor receptor tyrosine kinase inhibitor (EGFR‐TKI).

	Total		Non‐brain metastases		Brain metastases	
	aRR (95% CI)	*p*‐value	aRR (95% CI)	*p*‐value	aRR (95% CI)	*p*‐value
Hyperlipidemia	1.65 (1.01, 2.70)	.047	2.11 (1.07, 4.17)	.031	0.97 (0.46, 2.02)	.932
Hypertension	1.22 (0.68, 2.22)	.522	1.16 (0.54, 2.51)	.691	1.44 (0.69, 2.98)	.331
Diabetes mellitus	1.72 (0.91, 3.25)	.097	1.72 (0.76, 3.90)	.191	2.03 (0.98, 4.22)	.058
History of smoke	1.12 (0.66, 1.91)	.669	1.14 (0.54, 2.41)	.739	1.25 (0.64, 2.42)	.513
Heavy drinking	0.92 (0.28, 3.00)	.893	0.88 (0.15, 5.09)	.565	1.35 (0.53, 3.38)	.523
SBP, mmHg	1.01 (0.99, 1.03)	.326	1.02 (1.00, 1.04)	.020	0.99 (0.96, 1.02)	.548
DBP, mmHg	1.00 (0.98, 1.03)	.800	1.03 (1.00, 1.08)	.083	1.00 (0.96, 1.03)	.845
Radiotherapy	2.37 (1.51, 3.71)	.001	NA	–	2.03 (0.93, 4.44)	.078
IL‐2, pg/mL	1.30 (1.10, 1.53)	.002	1.08 (1.07, 1.53)	.006	1.63 (1.16, 2.27)	.004
IL‐4, pg/mL	1.43 (1.14, 1.81)	.003	1.44 (1.10, 1.88)	.008	1.52 (0.99, 2.32)	.051
IL‐6, pg/mL	1.00 (1.00, 1.01)	.653	1.00 (0.98, 1.01)	.328	1.00 (1.00, 1.00)	.060
IL‐10, pg/mL	1.31 (1.10, 1.60)	.003	1.30 (1.04, 1.61)	.019	1.48 (1.16, 1.90)	.002

*Note*: aRR adjust for: Age.

Abbreviations: CI, confidence interval; DBP, diastolic blood pressure; NA, not applicable; SBP, systolic blood pressure.

### 3.5 Possible mechanisms by which EGFR‐TKI enhances the risk of WMH progression

**FIGURE 4:**
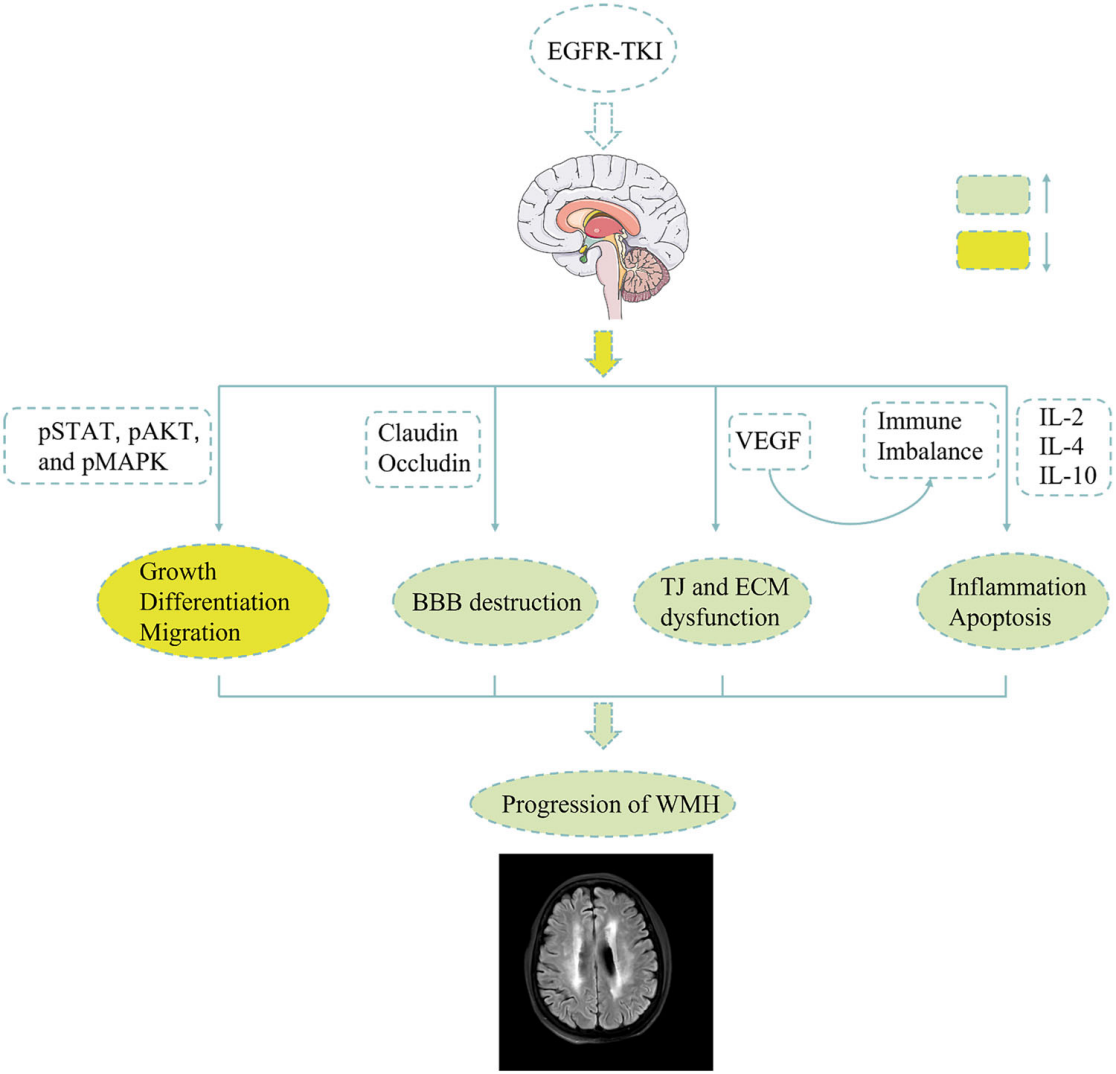
The possible pathways of white matter hyperintensity induced by long‐term epidermal growth factor receptor tyrosine kinase inhibitor (EGFR‐TKI) therapy. BBB, blood–brain barrier; ECM, extracellular matrix; IL, interleukin; pAKT, phosphor‐serine‐threonine kinase; pMAPK, phosphor‐mitogen‐activated protein kinase; pSTAT, phosphor‐signal transducer and activator of transcription; TJ, tight junction; VEGF, vascular endothelial growth factor.

We performed GO and KEGG pathway enrichment analysis to determine the relationship between age‐related periventricular lesions (GSE157363) and PC9 treated with EGFR‐TKI (GSE114647). The top 20 results of enrichment are shown in Figure S2A,B. The KEGG pathway contained nine common pathways including MAPK signaling pathway (hsa04010), oxytocin signaling pathway (hsa04921), cAMP signaling pathway (hsa04024), and so forth (Figure S2A). In addition, the DEGs were involved in nine common biological processes (Figure S2B), such as metal ion homeostasis (GO: 0055065), synaptic signaling (GO: 0099536), and extracellular matrix (GO: 0031012). As shown in the heatmap, genes that were enriched in the MAPK signaling pathway, oxytocin signaling pathway, and cAMP signaling pathway were upregulated in the PC9 treated with EGFR‐TKI (Figure S2C). Similarly, genes on the common signaling pathway showed elevated expression in the periventricular lesions dataset. Figure 4 shows the possible mechanism of white matter effects after long‐term administration of EGFR‐TKIs

## 4 DISCUSSION

In our study, there was a clear association between more than 2 years of EGFR‐TKI therapy and WMH progression in patients with NSCLC. After adjusting for sex, age, BMI, hyperlipidemia, hypertension, diabetes, brain metastases, and radiotherapy, the EGFR‐TKI group had an almost 2.78‐fold higher relative risk of increased WMH load compared to non‐EGFR‐TKI.

The pathophysiological mechanisms of the formation of WMH have not been fully elucidated, and ischemic demyelination, axonal loss, and gliosis can be observed histologically (K. W. Kim et al., 2008). According to the literature, EGF is expressed in the cortical plate during neural development and promotes axonal outgrowth of cortical neurons, and the EGFR pathway is associated with a variety of neuronal cellular events such as proliferation, differentiation, and apoptosis (Goldshmit et al., 2004; I.‐S. Kim et al., 2017). Therefore, long‐term EGFR‐TKI treatment use may result in brain abnormalities. Previous research has found an average annual increase of 0.6 mL of WMH in people between the ages of 60–70 years. We have found an annual increase of 2.7 mL/year in patients taking EGFR‐TKI (de Leeuw et al., 2001). A 7‐year follow‐up of patients with carotid artery stenosis revealed an increase in Fazekas scale scores in 21% of participants (Ihle‐Hansen et al., 2021). In our cohort, WMH progressed in 39 of 106 (36.7%) patients receiving EGFR‐TKI.

Several studies have demonstrated the role of Th2 inflammatory markers including IL‐4 and IL‐10 in intracerebral inflammation. Treatment with anti‐IL‐4 increased the number of mature oligodendrocytes and myelin proteins (Zanno et al., 2019). IL‐10 is considered an anti‐inflammatory factor, but it also indirectly responds to the severity of neuroinflammation (Sanchez‐Molina et al., 2021). IL‐2, reported to be toxic to oligodendrocytes and myelin, could play a role in the molecular cascade leading to white matter damage in periventricular leukomalacia (Kadhim et al., 2002).

In patients with brain metastases, radiation therapy is associated with a higher incidence of WMH. Radiation therapy could result in vascular injury. Increased permeability and disruption of the blood–brain barrier (BBB) characterize vascular injury. High‐dose focal radiation treatment induces endothelial cell deficiency, leading to vasogenic edema and ischemia via acid sphingomyelinase‐dependent apoptosis (Soussain et al., 2009). Hypoxia and the release of reactive oxygen radicals caused by tissue ischemia and vasogenic edema impaired cellular function. Radiation directly damages glial cells, resulting in impaired myelin formation, myelin sparing, reactive gliosis, and even coagulative necrosis and the formation of white matter cavities (Piao et al., 2015).

It is also commonly believed that WMH is caused by inadequate perfusion of small vessels. Many studies suggest that hypertension‐induced changes in large arteries can affect small arteries in a variety of ways, including small artery remodeling and arterial stiffness (Laurent & Boutouyrie, 2015; Stefanadis et al., 1995). In general, rising blood pressure causes arterial injury due to biomechanical fatigue of the elastic arterial wall (Lacolley et al., 2009). Individuals with higher SBP have less cerebrovascular tortuosity and branch number, resulting in white matter ischemia and WMH (Zhang et al., 2021).

Treatment with EGFR‐TKI may result in the growth of white matter hyperintensity through the pathways as shown in Figure 4. From the results of enrichment analysis, white matter lesions and EGFR‐TKI treatment overlap in multiple signaling pathways. The use of EGFR‐TKIs directly affects the MAPK signaling pathway. Studies have shown that MAPK is closely related to brain axonal and white matter remodeling (Fattah et al., 1991; Guo et al., 2020). The use of EGFR‐TKI results in decreased expression of tight junction proteins, which can lead to dysfunction of the BBB (Holcmann & Sibilia, 2015). Long‐term use of EGFR‐TKIs increases VEGF, which affects the stability of the BBB (Viloria‐Petit et al., 2001). Increased BBB permeability is associated with the progression of white matter hyperintensity. The release of VEGF also affects the immune microenvironment, causing an increase in inflammatory factors, neuroinflammation, and white matter damage (Proescholdt et al., 2002). As a result, EGFR‐TKI administration may have an adverse effect on the white matter brain structure through various pathways.

There were several limitations to this study. First, relatively small sample sizes may limit the reliability of conclusions. Second, there is no analysis of neurological performance. Third, no healthy subjects were included in this retrospective study for comparison. Furthermore, for a one‐point increase on the Fazekas scale, the change must be of a certain magnitude, limiting the detectability of change in WMH. Due to the different purposes of the study, the selected transcriptomic data do not represent the actual pathology. Therefore, prospective studies as well as in vitro and in vivo research are required to confirm the risks of anti‐EGFR therapy.

## 5 CONCLUSION

In patients with NSCLC treated with EFGR‐TKI, the risk of developing brain WMH is increased by nearly threefold. Insidious changes in WMH may lead to poor outcomes such as cognitive decline. Furthermore, IL‐2, IL‐4, and IL‐10 may act as independent predictors to increase the WMH load in patients with NSCLC. Lastly, genes enriched in signaling pathways such as MAPK may be related to WMH progression. Future studies are needed to confirm these results.

## AUTHOR CONTRIBUTIONS

Experimental design: Bo Hu, Yifan Zhou, Kunyu Yang, Jiang Chang. Data collection: Rui Meng, Junjie Jiang, Yan Luo, Xiaolin Deng, Yanan Li, Yan Wan, Jiehong Wu, Shengcai Chen, Sibo Yang. Data analysis and interpretation: Bo Hu, Yifan Zhou, Hang Yang, Rui Meng, Junjie Jiang, Quanwei He, Huijuan Jin. Manuscript drafting and revisions: Bo Hu, Yifan Zhou, Hang Yang, Rui Meng, David Wang, Kunyu Yang.

## CONFLICT OF INTEREST STATEMENT

The authors declare no conflicts of interest.

### PEER REVIEW

The peer review history for this article is available at https://publons.com/publon/10.1002/brb3.3326

## Supporting information

Supporting Information

## Data Availability

The data that support the findings of this study are available from the corresponding author upon reasonable request.
