# Supplementary material for: Association of white matter hyperintensities with long‐term EGFR‐TKI treatment and prediction of progression risk
Source: Brain Behav. 2023 Dec 6;13(12):e3326. doi: 10.1002/brb3.3326 (PMC10726800; doi:10.1002/brb3.3326)
Supplement: Supplementary file 1 — Supporting Information [file BRB3-13-e3326-s001.docx]

Supplementary figure 1 Comparison of Proportions of Fazekas Scores in Total, Periventricular, and Deep White Matter Hyperintensities (WMH) Scales Among the EGFR-TKI and Non-EGFR-TKI Group

Supplementary Figure 2 Identification of the possible mechanisms using transcriptome analysis. (A) KEGG pathway enrichment analysis for Differentially expressed genes (DEGs) of GSE11464 and GSE157363 datasets using Metascape. The top 9 pathways were the common pathways. (B) GO enrichment analysis in biological processes for DEGs. The top 9 processes were the common enriched pathways. (C) Heatmaps of genes enriched in MAPK signaling pathway, Oxytocin signaling pathway and cAMP signaling pathway.

Supplementary figure 1


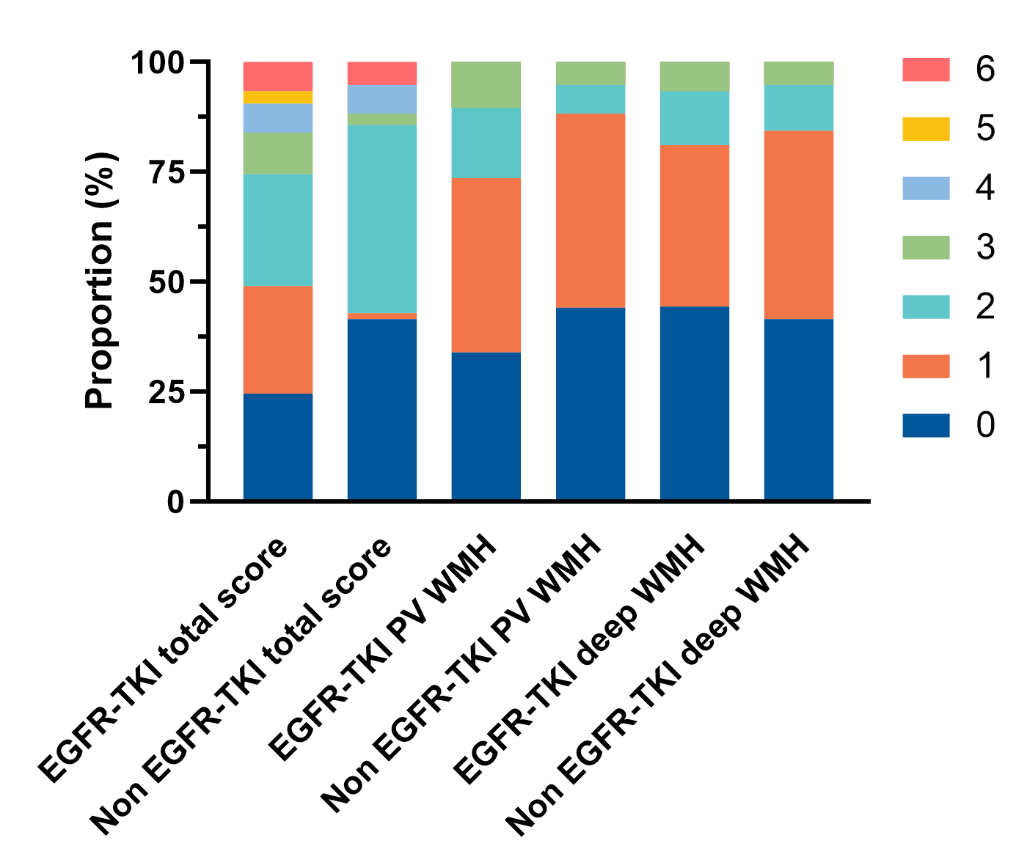


Supplementary figure 2


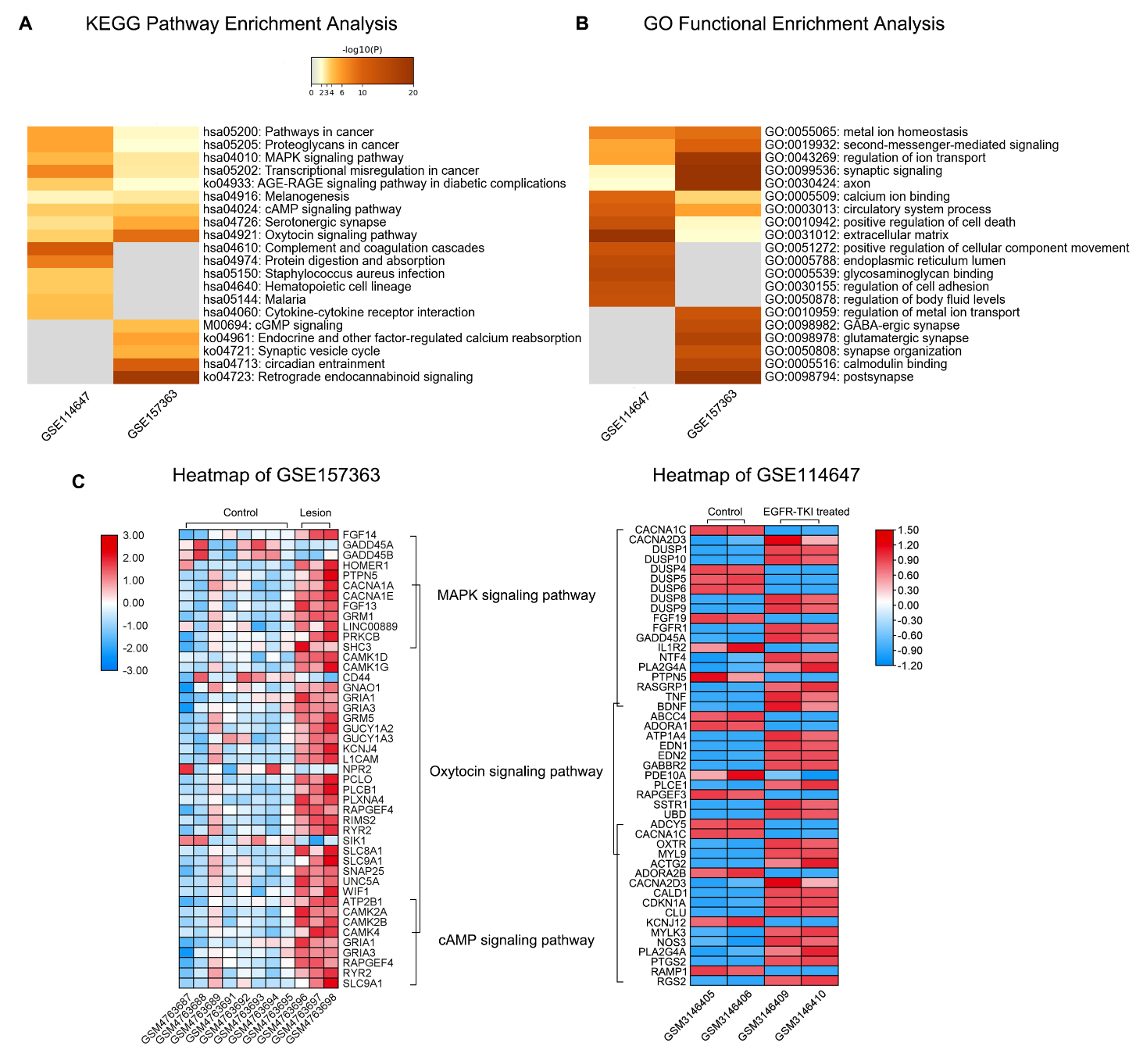


Supplemental Table 1. Clinical Risk Factor Variables and EGFR-TKI treatment of the Cohort of Patients Identified with NSCLC and Their Associations with Increased White Matter Hyperintensities (WMH) Load.

|  | aRR (95%CI) | *p*-value |
| --- | --- | --- |
| EGFR-TKI | 2.52 (1.37-4.68) | 0.003 |
| Hyperlipidemia | 1.14 (0.47-2.76) | 0.778 |
| Hypertension | 0.89 (0.36-2.21) | 0.803 |
| Diabetes | 0.77 (0.23-2.57) | 0.665 |
| Brain metastases | 1.91 (1.14-3.21) | 0.014 |
| Radiotherapy | 2.27 (1.36-3.80) | 0.002 |

adjust for: Sex; Age, BMI, History of smoke, Heavy drinking

Supplemental Table 2. Effect Size Between Laboratory Biomarker and White Matter Hyperintensities (WMH)

|  | β | *p*-value |
| --- | --- | --- |
| Total |  |  |
| CEA, ng/mL | 0.96 | 0.547 |
| CA19-9, ng/mL | 1 | 0.756 |
| IL-2, pg/mL | 1.12 | 0.012 |
| IL-4, pg/mL | 1.17 | 0.005 |
| IL-6, pg/mL | 0.99 | 0.948 |
| IL-10, pg/mL | 1.04 | 0.029 |
| EGFR-TKI |  |  |
| CEA, ng/mL | 0.96 | 0.632 |
| CA19-9, ng/mL | 1 | 0.018 |
| IL-2, pg/mL | 1.13 | 0.001 |
| IL-4, pg/mL | 1.16 | 0.001 |
| IL-6, pg/mL | 1 | 0.795 |
| IL-10, pg/mL | 1.24 | 0.001 |
| Non-EGFR-TKI |  |  |
| CEA, ng/mL | 1 | 0.724 |
| CA19-9, ng/mL | 0.93 | 0.681 |
| IL-2, pg/mL | 0.79 | 0.623 |
| IL-4, pg/mL | 1.09 | 0.037 |
| IL-6, pg/mL | 0.78 | 0.485 |
| IL-10, pg/mL | 0.62 | 0.586 |

Adjust for: Sex, Age, BMI, Hyperlipidemia, Hypertension, Diabetes, History of smoke, Heavy drinking
